# Patterns of non-communicable disease and injury risk factors in Kenyan adult population: a cluster analysis

**DOI:** 10.1186/s12889-018-6056-7

**Published:** 2018-11-07

**Authors:** Tilahun Nigatu Haregu, Frederick M Wekesah, Shukri F Mohamed, Martin K Mutua, Gershim Asiki, Catherine Kyobutungi

**Affiliations:** 1 African Population and Health Research Center (APHRC), 2nd Floor APHRC Campus, Manga Close Off Kirawa Road, P.O. Box 10787, Kitisuru, Nairobi GPO 00100 Kenya; 2 Julius Global Health, Julius Center for Health Sciences and Primary Care, University Medical Center, Utrecht University, Utrecht, The Netherlands; 3 Division of Health Sciences, Warwick Medical School, University of Warwick, Coventry, UK; 4 Non-communicable Diseases Unit, University of Melbourne, Melbourne, Australia

**Keywords:** Non-communicable disease, Injury, Risk factor, Cluster analysis, STEPS, Kenya

## Abstract

**Background:**

Non-communicable diseases and unintentional injuries are emerging public health problems in sub-Saharan Africa. These threats have multiple risk factors with complex interactions. Though some studies have explored the magnitude and distribution of those risk factors in many populations in Kenya, an exploration of segmentation of population at a national level by risk profile, which is crucial for a differentiated approach, is currently lacking. The aim of this study was to examine patterns of non-communicable disease and injury risk through the identification of clusters and investigation of correlates of those clusters among Kenyan adult population.

**Methods:**

We used data from the 2015 STEPs survey of non-communicable disease risk factors conducted among 4484 adults aged between 18 and 69 years in Kenya. A total of 12 risk factors for NCDs and 9 factors for injury were used as clustering variables. A K-medians Cluster Analysis was applied. We used matching as the measure of the similarity/dissimilarity among the clustering variables. While clusters were described using the risk factors, the predictors of the clustering were investigated using multinomial logistic regression.

**Results:**

We have identified five clusters for NCDs and four clusters for injury based on the risk profile of the population. The NCD risk clusters were labelled as cluster hypertensives, harmful users, the hopefuls, the obese, and the fat lovers. The injury risk clusters were labelled as helmet users, jaywalkers, the defiant and the compliant. Among the possible predictors of clustering, age, gender, education and wealth index came out as strong predictors of the cluster variables.

**Conclusion:**

This cluster analysis has identified important clusters of adult Kenyan population for non-communicable disease and injury risk profiles. Risk reduction interventions could consider these clusters as potential target in the development and segmentation of a differentiated approach.

## Background

Non-communicable diseases (NCDs) cause more deaths globally than all other causes combined together [1, 2]. In 2012, about 38 million people died from NCDs, and the number of deaths is projected to reach 52 million by the year 2030 [1–5]. Cardiovascular diseases (CVDs), cancers, chronic respiratory diseases and diabetes comprise 80% of NCDs. The shift in the global burden of disease from communicable diseases to NCDs is attributed to population growth and the increased average age of the world’s population, combined with the decreasing age-, sex- and cause-specific death rates [6].

NCDs are caused by multiple risk factors which interact in a complex way [7]. Many of the risk factors for NCDs are related to lifestyle and are therefore modifiable. These modifiable risk factors include physical inactivity, low fruit and vegetable intake, unhealthy diet and high cholesterol intake. Physiological risk factors for NCDs include overweight and obesity [7, 8].

Achieving the 25*25 target, which is the reduction of premature mortality from four main NCDs—cardiovascular diseases, chronic respiratory diseases, cancers, and diabetes—by 25% from 2010 levels by 2025 [7] will very much depend on achieving the risk factor target on the key risk factors for NCDs (tobacco and alcohol use, salt intake, obesity, and raised blood pressure and glucose) [7].

As the risk factors for many of the common NCDs are shared, the likelhood of their co-occurrence is high. Thus, studies of single risk factors or prevalence of individual risk factors will miss the complex interaction among the risk factors. For a better understanding of risk profiles of a population, the whole set of risk factors should be considered. Hence, there is a need for approaches that consider common risk factors together to describe risk profile of the population.

This study sought to investigate patterns of NCD risk factors, hence profiles of the Kenyan population based on the clustering of these risk factors. Different segments of the population experience, or are exposed to different risk factors and therefore have different risk profiles, and will require targeted approaches and interventions in mitigating these risk factors for the prevention of NCDs.

## Methods

The Kenya 2015 STEPS survey was a cross-sectional household survey that was carried out in Kenya from April to June 2015,targeting individuals aged between 18 and 69 years. The survey used the fifth national sample surveys and evaluation programme (NASSEP V) sampling frame from the Kenya National Bureau of Statistics, developed using the enumeration areas generated from the 2009 Kenya population and Housing census. The sample size was determined to be 6000 to allow for national estimates as per sex and residence (rural or urban).

A three stage cluster sample design was used. In the first stage, 200 clusters (100 urban and 100 rural) were selected. In the second stage, a uniform sample of 30 households from the listed households in each cluster, while in the third stage, one individual was randomly selected from all eligible listed household members.

### Data collection

Socio-demographic and behavioral information was collected in step 1, physical measurements such as height, weight and blood pressure were collected in step 2 while biochemical measurements for blood glucose and cholesterol were taken in step 3 with respondents in a fasting state.

The survey focussed on the four main behavioural risk factors of NCDs: tobacco use, harmful alcohol consumption, unhealthy diet and lack of physical activity; and the four key physiological risk factors for NCDs:overweight and obesity, raised blood pressure, raised blood lipids and raised blood glucose. The survey questionnaire was adapted from the WHO STEPS instrument [9], with information being gathered in three sequential steps. Step one involved asking questions on demographic information such as age, sex, marital status, education and occupation, housing and social amenities as well as dietary history on salt, sugar, fat, fruits and vegetable intakes. Data collection was through a personal digital assistant (PDA) loaded with eSTEPS software provided by WHO.

Twenty multidisciplinary teams (supervisor, two research assistants, a clinician and laboratory technologist) were involved in data collection after undergoing a six day training on survey background, sampling method, questioning techniques, PDA use and ethical procedures.

### Key variables

Twelve traditional non-communicable disease risk factors and nine risk factors for injuries were used in our analysis. These measures were both self-reported and objectively measured. The inclusion of these risk factors was based on availability of complete data for the study population. The cut-off points for these variables were based on international recommendations [10–13].

### Risk variables for NCDs and injury

#### NCD risk variables

Inadequate fruit/vegetable intake, high sugar intake, insufficient physical activity, harmful alcohol use, tobacco use, excessive sitting time, general obesity, central obesity, high blood sugar, high salt consumption, high fat intake, and increased blood pressure.

#### Injury risk factors

Didn’t use seatbelt, didn’t use helmet, involved in traffic crash, had accidental injury, inappropriate road crossing, driving under influence of alcohol, was a passenger of drunk driver, involved in violence, and substance use/e.g. khat.

### Data management and analysis

We used Stata 14.1 (Stata Corporation, College Station, TX) to analyse the data. Analysis was restricted to individuals with complete data on the key analytic variables listed above. Those with missing values were excluded from the analysis.

#### Cluster identification

For both categories of risk factors, the variables were recoded as 0 (low risk) and 1 (higher risk). Given the nature of the data, binary data, we used K-median cluster analysis approach. We used matching as a measure of distance of proximity. We used the scree plot to determine the ideal number of clusters.

#### Cluster characterization

The distribution of the risk factors across the clusters was examined to characterize the clusters based on the risk profile. Clusters were named based on their unique dominant risk profiles. The background characteristics of participants in each cluster were also summarized using proportions and the associations were tested using chi-square statistics.

#### Predictors of cluster distribution

Predictors of the cluster distributions were examined using logistic regression models. The background characteristics included in the model were age, gender, education, employment, residence, wealth index, and marital status. Results of this are presented in tables.

### Ethical considerations

Written informed consent was obtained from every participant. Personal identifiers were delinked from the data by coding and the consent forms that contained personal identifiers were stored separately from the coded data. The data collection team was trained on ethical procedures and appropriate data collection techniques.

## Results

### Characteristics of study population

In total 4484 adults aged between 18 and 69 years were included in the study with nearly an equal representation women and men (51.3% versus 48.8%), and about half were young people aged 18–29 years, 65.5% married, 61.9% were rural residents, 12.6% had no formal education, 18.9% were classified as poorest and 23.4% richest and up to 40.1% were unemployed (Table 1).

**Table 1:** Socio-demographic characteristics

Characteristics	Number	Percent
Sex		
Female	2298	51.3
Male	2186	48.8
Age groups		
18–29	2062	46.0
30–39	1045	23.3
40–49	695	15.5
50–59	443	9.9
60–69	239	5.3
Marital status		
Not married	1039	23.2
Married	2938	65.5
Formerly married	507	11.3
Residence		
Rural	2776	61.9
Urban	1708	38.1
Education level		
No formal education	563	12.6
Primary education	2043	45.6
Secondary and above	1877	41.9
Household wealth status		
Poorest	848	18.9
Second	937	20.9
Middle	818	18.3
Fourth	832	18.6
Richest	1049	23.4
Occupation		
Unemployed	1799	40.1
Employed	2685	59.9

#### Cluster analysis of NCD risk factors

Using the 12 risk variables, the optimum number of clusters was found to be five. The distribution of the risk variables across the clusters is shown in Table 2 below.

**Table 2:** Distribution of specific risk factors by clusters

SN	NCD Risk Variables	Cluster 1	Cluster 2	Cluster 3	Cluster 4	Cluster 5	Total
1	Inadequate Fruit/vegetables	99.4	100	99.7	99.8	99.8	99.8
2	High sugar intake	12.2	11.8	11.5	16.9	17.0	13.9
3	Insufficient physical activity	7.8	4.8	9.2	11.9	4.7	8.8
4	Excessive alcohol use	10.6	*98.5*	6.6	5.7	0.0	11.8
5	Tobacco use	9.1	*65.8*	8.2	3.3	12.6	12.3
6	Excessive sitting time	25.3	20.6	24.8	23.9	15.1	22.1
7	General obesity	9.5	5.5	8.4	*99.3*	6.5	31.4
8	Central obesity	25.1	10.7	14.5	*99.2*	11.1	36.8
9	High blood sugar	13.5	9.9	9.1	15.7	6.6	10.9
10	High salt consumption	12.9	*34.6*	21.0	18.5	18.7	19.6
11	High fat intake	38.1	80.1	0.0	29.0	*100*	41.5
12	Increased blood pressure	*100*	27.9	0.0	42.0	4.3	27.6
	Cluster size	549	272	1196	1024	922	3963
	% of total	14.0	7.0	30.0	26.0	23.0	100
	Suggested cluster name	Hypertensives	Harmful users	Hopefuls	The obese	Fat lovers	

*NCD* = Non-Communicable diseases; The clusters are groups of study participants with similar pattern of NCD risk factors; All the values for risk factors are percentages. The *italicised* proportions characterise the cluster

As displayed in the Table 2, participants in cluster 1 were all with hypertension. We labelled this cluster as “hypertensives.” Participants in cluster 2 had high rates of harmful use of alcohol, tobacco use and salt consumption as compared to the rest of the clusters. We labelled this cluster as “harmful users.” Participants in the fourth cluster had highest rates of general and abdominal obesity. These were labelled as “the obese.” Those in the fifth cluster had the highest rate of high fat consumption and thus were labelled as “fat lovers.” Participants in the third cluster have no extreme risk and were labelled as the “hopefuls.” Inadequate fruit and vegetable consumption was universal across all five clusters. Similarly, physical inactivity was not common in all clusters.

#### Profile of the NCD risk clusters

As compared to the other clusters, the hopefuls and fat lovers are younger. The mean (SD) ages were 33.9 (12.3) and 34.6 (12.4) years respectively. The mean ages for hypertensives, harmful users and the obese were 43.2 (14.8), 40.9 (12.8), and 41.2 (12.6) years, respectively. As to gender, majority of the harmful users (87%) were male. On the other hand, more than three quarters (78%) of the obese were female. Majority of the obese were urban residents while the fat lovers were rural residents. The hopefuls are equally distributed between rural and urban areas. A little more than half of the hypertensives and harmful users lived in rural areas. Details are shown in Table 3.

**Table 3:** Clusters of NCD risk factors by background variables

SN	NCD Risk Variables	Hypertensives	Harmful users	Hopefuls	The obese	Fat lovers	*p*-value
1	Age						
	18–29	23.9	19.5	43.9	20.0	40.1	< 0.001
	30–44	29.3	43.8	36.0	42.8	39.8	
	45–59	27.0	24.6	14.9	26.1	14.4	
	60–69	19.9	12.1	5.3	11.1	5.6	
2	Sex						
	Female	53.7	12.9	58.8	78.1	56.3	< 0.001
	Male	46.3	87.1	41.2	21.9	43.7	
3	Residence						
	Rural	55.9	57.0	50.1	41.4	60.0	< 0.001
	Urban	44.1	43.0	49.9	58.6	40.0	
4	Education						
	No schooling	20.0	13.2	18.6	11.4	16.5	< 0.001
	Primary incomplete	21.0	35.3	24.2	20.3	29.0	
	Primary complete	32.6	28.3	27.8	33.4	35.1	
	Secondary+	26.4	23.2	29.4	34.9	19.4	
5	Employment						
	Employed	45.9	30.2	43.1	33.7	47.2	< 0.001
	Unemployed	54.1	69.9	56.9	66.3	52.8	
6	Wealth index						
	Poorest	20.0	25.7	24.5	8.1	26.6	< 0.001
	Second	23.0	24.6	18.2	12.9	27.3	
	Third	24.4	19.9	17.1	21.2	20.0	
	Fourth	18.4	18.0	19.2	23.7	17.7	
	Richest	14.2	11.8	20.9	34.1	8.5	
7	Marital status						
	Not in Union	34.2	37.5	36.2	27.1	30.4	< 0.001
	Union	65.8	62.5	63.8	72.9	69.6	

*NCD* = Non-communicable diseases; Values in the clusters are percentages

The proportion of people in the hopeful and obese groups increased with educational status. The harmful users and the obese were dominated by the unemployed (70% and 66%, respectively). Analysis of wealth index among the clusters showed that the proportion of participants in the obese cluster increased linearly with wealth index.

### Predictors of the NCD clusters

For the NCD risk clusters, in multivariate analysis, higher age was found to be the predictor for the hypertensives. Being male was the strongest factor associated with belonging to the harmful users’ cluster. We also found that wealth was strongly associated with the obese cluster. Age, educational status and wealth index were associated with the hopefuls cluster. Details are shown in Table 4 below.

**Table 4:** Predictors of NCD risk clusters

	Hypertensives		Harmful users		Hopefuls		The obese		Fat lovers	
	OR	(95% CI)	OR	(95% CI)	OR	(95% CI)	OR	(95% CI)	OR	(95% CI)
Age										
18–29 years (reference)	1.00		1.00		1.00		1.00		1.00	
30–44 years	1.11	(0.85, 1.43)	2.11	(1.44, 3.07)	0.59	(0.50, 0.70)	2.76	(2.22, 3.42)	0.67	(0.56, 0.81)
45–59 years	2.09	(1.59, 2.73)	2.54	(1.67, 3.88)	0.4	(0.32, 0.49)	4.37	(3.42, 5.59)	0.4	(0.32, 0.51)
60–69 years	3.62	(2.66, 4.94)	2.43	(1.47, 4.01)	0.25	(0.18, 0.34)	5.25	(3.82, 7.21)	0.3	(0.21, 0.42)
Sex										
Female (reference)	1.00		1.00		1.00		1.00		1.00	
Male	1.44	(1.18, 1.76)	14.3	(9.74, 21.03)	1.08	(0.93, 1.25)	0.19	(0.15, 0.23)	1.37	(1.16, 1.61)
Marital status										
Not in union (reference)	1.00		1.00		1.00		1.00		1.00	
In Union	0.92	(0.74, 1.14)	0.51	(0.37, 0.70)	0.85	(0.72, 1.00)	1.62	(1.33, 1.97)	1.1	(0.92, 1.32)
Residence										
Rural (reference)										
Urban	0.94	(0.76, 1.16)	0.96	(0.71, 1.31)	1.13	(0.96, 1.33)	0.99	(0.82, 1.20)	1.02	(0.85, 1.22)
Education										
No schooling (reference)	1.00		1.00		1.00		1.00		1.00	
Primary incomplete	0.76	(0.56, 1.04)	1.43	(0.91, 2.25)	0.71	(0.56, 0.90)	1.21	(0.90, 1.63)	1.13	(0.88, 1.46)
Primary complete	1.08	(0.79, 1.47)	0.87	(0.54, 1.40)	0.56	(0.44, 0.72)	1.48	(1.10, 1.99)	1.14	(0.87, 1.48)
Secondary +	1.14	(0.81, 1.61)	0.79	(0.47, 1.33)	0.67	(0.52, 0.88)	1.63	(1.18, 2.24)	0.86	(0.64, 1.17)
Employment										
Unemployed (reference)	1.00		1.00		1.00		1.00		1.00	
Employed	0.77	(0.63, 0.95)	0.96	(0.70, 1.31)	1.04	(0.88, 1.22)	1.35	(1.12, 1.62)	0.86	(0.73, 1.03)
Wealth index										
Poorest (reference)	1.00		1.00		1.00		1.00		1.00	
Second quintile	1.22	(0.90, 1.66)	0.8	(0.54, 1.20)	0.75	(0.59, 0.94)	1.65	(1.19, 2.27)	1.04	(0.83, 1.32)
Third quintile	1.26	(0.93, 1.71)	0.68	(0.45, 1.05)	0.7	(0.55, 0.89)	2.94	(2.15, 4.02)	0.71	(0.55, 0.91)
Fourth quintile	1	(0.71, 1.41)	0.61	(0.38, 0.97)	0.72	(0.56, 0.93)	4.63	(3.33, 6.45)	0.54	(0.41, 0.71)
Richest	0.82	(0.55, 1.22)	0.42	(0.24, 0.74)	0.74	(0.56, 0.99)	9.43	(6.57, 13.53)	0.23	(0.16, 0.33)
Adults in household										
One (reference)	1.00		1.00		1.00		1.00		1.00	
Two adults	0.98	(0.79, 1.23)	1	(0.73, 1.37)	1.02	(0.86, 1.20)	1.01	(0.84, 1.22)	0.95	(0.79, 1.13)
Three or more adults	0.96	(0.73, 1.27)	0.51	(0.33, 0.80)	1.41	(1.15, 1.74)	1.07	(0.83, 1.37)	0.66	(0.52, 0.84)
Constant	0.11	(0.08, 0.16)	0.02	(0.01, 0.03)	1.22	(0.95, 1.57)	0.03	(0.02, 0.05)	0.63	(0.48, 0.84)

#### Cluster analysis of injury risk factors

Using the same cluster analysis approach for the nine injury risk factors, the optimum number of clusters was found to be four. A total of 3981 participants were included in this cluster analysis. The distribution of the injury risk factors is shown in Table 5.

**Table 5:** Distribution of injury risk factors by clusters

SN	NCD Risk Variables	Cluster 1	Cluster 2	Cluster 3	Cluster 4	Total
1	Didn’t use seatbelt	25.1	0.0	100	0.0	55.3
2	Didn’t use helmet	0.0	100	100	100	67.1
3	Involved in traffic crash	5.6	6.0	4.5	6.4	5.3
4	Had injury	9.3	12.0	12.2	16.7	11.4
5	Inappropriate road crossing	76.5	100	91.4	0.0	84.1
6	Driving under influence of alcohol	2.9	2.7	3.0	2.5	2.9
7	Was a passenger of drunk driver	9.8	12.6	12.9	18.7	12.1
8	Involved in violence	2.9	3.9	4.6	5.9	3.9
9	Substance use	6.9	5.7	6.4	5.9	6.4
	Cluster size	1310	1019	1449	203	3981
	% of total	33.0	26.0	36.0	5.0	100
	Suggested cluster name	Helmet users	Jaywalkers	The defiant	The compliant	

*NCD* = Non-Communicable diseases; the clusters are groups of study participants with similar pattern of NCD risk factors; All the values for risk factors are percentages

While characterizing the clusters by risk factors we found that participants in cluster 1 had considerable use of helmets when they use motorcycle, cycle or scooter. This group was labelled as “Helmet users.” Those in the second cluster were known for inappropriate road crossing and are labelled as “jaywalkers.” All the participants in the third cluster didn’t use seatbelt when they had to. We labelled this group as “the defiant.” Lastly, those in the fourth cluster, had remarkable level of seatbelt use and they did appropriate road crossing. We labelled this cluster as “the compliant.”

#### Profile of injury clusters

The average age decreased modestly as one goes from helmet users to the compliant though the differences were not significant. Education of the participants was found to be an important factor in the profiling of the clusters. We found that the proportion of participants in the compliant cluster increases with their educational level. Close to 60% of the helmet users had completed at least primary education. On the contrary, about 60% of the jaywalkers had a similar educational level. While more than 40% of the seatbelt users and the compliant were on the higher side of wealth index, 46% of the defiant were in the lower wealth index category. Surprisingly, 44% of the jaywalkers were also within the higher wealth index categories. Profile of injury clusters is summarized in Table 6.

**Table 6:** Clusters of injury risk factors by background variables

SN	NCD Risk Variables	Helmet users	Jaywalkers	The defiant	The compliant	Chi-square (P-value)
1	Age					
	18–29	30.9	32.2	36.3	37.4	Chi = 16.6 *P* = 0.055
	30–44	39.2	40.7	36.2	38.4	
	45–59	20.0	19.3	18.2	15.3	
	60–69	9.9	7.8	9.3	8.9	
2	Sex					
	Female	54.4	58.6	65.1	58.1	Chi = 33.6 *P* = 0.000
	Male	45.7	41.4	34.9	41.9	
3	Residence					
	Rural	54.1	49.2	52.3	43.8	Chi = 10.7 *P* = 0.013
	Urban	46.0	50.8	47.7	56.2	
4	Education					
	No schooling	17.1	9.3	26.4	6.4	Chi = 155.6 *P* = 0.000
	Primary incomplete	24.7	25.1	24.0	26.1	
	Primary complete	31.0	34.0	28.3	30.1	
	Secondary+	27.2	31.6	21.3	37.4	
5	Employment					
	Employed	38.5	37.6	46.5	34.0	Chi = 30.7 *P* = 0.000
	Unemployed	61.5	62.4	53.5	66.0	
6	Wealth index					
	Poorest	20.4	14.0	27.7	12.3	Chi= *P* = 0.000
	Second	18.4	22.0	19.6	22.2	
	Third	19.6	19.6	19.2	16.8	
	Fourth	20.2	20.9	19.5	20.7	
	Richest	21.5	23.5	14.0	28.1	
7	Marital status					
	Not in Union	29.9	31.9	32.0	35.0	Chi = 108.1 *P* = 0.383
	Union	70.2	68.1	68.0	65.0	

*NCD* = Non-Communicable diseases; Values in the clusters are percentages

### Predictors of the injury clusters

In the injury clusters, age, education and wealth were negatively associated with the likelihood of an individual to belong to the defiant group. Educational status was also a predictor of the compliant cluster, but also the jaywalkers’ cluster. Richest groups had high level of helmet use as compared to others. Predictors of injury clusters is displayed in Table 7.

**Table 7:** Predictors of the injury clusters

	Helmet users		Jaywalkers		The defiant		The compliant	
	OR	(95% CI)	OR	(95% CI)	OR	(95% CI)	OR	(95% CI)
Age								
18–29 (reference)	1.00		1.00		1.00		1.00	
30–44	1.14	(0.96, 1.34)	1.22	(1.02, 1.46)	0.76	(0.65, 0.90)	0.92	(0.65, 1.30)
45–59	1.23	(1.00, 1.50)	1.25	(1.01, 1.56)	0.71	(0.58, 0.87)	0.79	(0.50, 1.23)
60–69	1.31	(1.01, 1.70)	1.19	(0.89, 1.61)	0.64	(0.50, 0.83)	1.23	(0.71, 2.15)
Sex								
Female (reference)	1.00		1.00		1.00		1.00	
Male	1.38	(1.20, 1.59)	0.93	(0.80, 1.09)	0.79	(0.68, 0.92)	0.89	(0.66, 1.21)
Marital Status								
Not in union (reference)	1.00		1.00		1.00		1.00	
In union	1.10	(0.93, 1.29)	0.94	(0.79, 1.12)	0.95	(0.81, 1.11)	1.02	(0.73, 1.42)
Residence								
Rural (reference)	1.00		1.00		1.00		1.00	
Urban	0.72	(0.62, 0.85)	0.95	(0.80, 1.12)	1.40	(1.19, 1.64)	1.14	(0.81, 1.61)
Education								
No schooling (reference)	1.00		1.00		1.00		1.00	
Primary incomplete	1.05	(0.84, 1.32)	2.11	(1.60, 2.79)	0.52	(0.42, 0.64)	2.97	(1.55, 5.69)
Primary complete	1.04	(0.82, 1.31)	2.32	(1.75, 3.07)	0.49	(0.39, 0.61)	2.68	(1.38, 5.20)
Secondary +	0.94	(0.73, 1.22)	2.51	(1.85, 3.39)	0.46	(0.36, 0.59)	3.75	(1.88, 7.47)
Employment								
Unemployed (reference)	1.00		1.00		1.00		1.00	
Employed	1.00	(0.86, 1.17)	0.98	(0.83, 1.15)	0.99	(0.85, 1.15)	1.13	(0.81, 1.58)
Wealth Index								
Poorest (reference)	1.00		1.00		1.00		1.00	
Second	0.93	(0.74, 1.17)	1.41	(1.09, 1.82)	0.79	(0.63, 0.98)	1.38	(0.82, 2.34)
Third	1.12	(0.89, 1.42)	1.25	(0.96, 1.62)	0.78	(0.62, 0.97)	1.01	(0.57, 1.76)
Fourth	1.24	(0.97, 1.59)	1.31	(0.99, 1.73)	0.67	(0.53, 0.85)	1.06	(0.59, 1.88)
Richest	1.59	(1.20, 2.11)	1.52	(1.12, 2.06)	0.43	(0.32, 0.56)	1.28	(0.69, 2.36)
Adults in household								
One (reference	1.00		1.00		1.00		1.00	
Two	0.97	(0.83, 1.14)	1.10	(0.93, 1.31)	1.01	(0.86, 1.18)	0.76	(0.54, 1.06)
Three or more	0.98	(0.80, 1.21)	1.03	(0.83, 1.29)	1.01	(0.83, 1.24)	0.92	(0.60, 1.40)
Constant	0.36	(0.29, 0.47)	0.12	(0.09, 0.16)	1.69	(1.34, 2.14)	0.02	(0.01, 0.04)

## Discussion

The STEPS survey is the first countrywide population based NCD survey in Kenya and has provided important insights into the burden of NCD and injury risk profiles of both rural and urban populations. Cluster analysis was employed to determine patterns of NCD and injury risks and this segmented the population into five heterogeneous NCD risk clusters and four injury risk clusters. Two of the NCD risk clusters named fat lovers (23%) and harmful users (7%) demonstrated patterns consistent with three known behavioural NCD risk factors- unhealthy diet, tobacco smoking and harmful use of alcohol, and two NCD risk clusters referred to as the obese (26%) and the hypertensive (14%) fell in the physiological NCD risk group. One cluster had no extreme NCD risk. However, in all clusters fruit and vegetables consumption was way below the recommended five servings per day and physical inactivity was not common.

These findings are consistent with literature from rural and urban settings in Kenya highlighting that the burden of NCDs is driven by all the known behavioural and physiological NCD risk factors but not physical inactivity [14–16]. Recent publications from other countries in East Africa have revealed similar findings of dietary habits characterised by poor consumption of fruits and vegetables and a high consumption of fats and carbohydrate amidst adequate physical activity [17, 18], a pattern typical of an early phase of nutrition transition [19].

Our study has identified distinct population groups with prevalent NCD risk factors for targeted interventions. It is interesting to note that the smallest NCD risk cluster represents tobacco consumption, harmful alcohol consumption and excessive salt use. The lower frequency of harmful alcohol use and tobacco smoking may be a reflection of the relative success in the development and implementation of policies addressing the WHO “best buy” interventions for NCD prevention. These policies should ideally include measures to reduce common NCD risk factors such as tobacco use, unhealthy diet, physical inactivity and the harmful use of alcohol – that would deliver the greatest benefit in reducing population level risks in a cost-effective manner [20].

A recent NCD prevention policy review for Kenya revealed a fairly better formulated tobacco control policy addressing all WHO “best buy” interventions such as tax increases, bans on tobacco advertising, and warnings on the dangers of tobacco; a weak alcoholic drinks control act (ADCA) addressing some of the “best buy” interventions including taxation and restriction to alcohol access; and a deficient food and nutrition policy not adequately addressing “best buy” interventions for unhealthy diet [21]. Although physical activity policies are not given priority, no cluster emerged with physical inactivity as the main risk factor because most people are active through work and travel other than recreation [22].

For injuries, 62% of the population was classified into two high risk injury clusters referred to as the defiant (36%) for not using seatbelts and jaywalkers (26%) because of inappropriate road crossing. The remaining two clusters which were low risk included helmet users (33%) and the compliant (5%) who used belts consistently and crossed roads appropriately. A recent survey conducted in five regional referral hospitals in Kenya showed that road traffic accidents were the most common injury among patients admitted in the emergency department and this is consistent with the clustering of risk factors at population level in this study [23]. Two other studies in Kenya have also revealed that among road traffic injuries, passengers in public transport vehicles followed by pedestrians were most involved [24, 25]. These accidents could have occurred because of non-compliance with belt use or jaywalking (inappropriate road crossing) reported in our study.

Identification of demographic characteristics associated with NCD risk clusters and the injury risk clusters is essential for programming successful primary preventive measures. We therefore profiled the NCD and injury risk clusters to inform differentiated prevention and care services. The factors that stood out as independent predictors of NCD risk clusters were; age, gender, education, wealth and living arrangements. Hypertension, harmful use of alcohol or salt and tobacco smoking, and obesity increased with age while fat consumption reduced with age. Men were more likely to be hypertensive, harmful users and fat lovers, while women were more likely to be obese.

The gender and age association with NCD risk has been well established before in Kenya [14]. An interesting finding in relation to age is the high consumption of fats by younger people. This may be explained by growing westernization of diet that young people are quickly adapting to and it is often observed in the early phase of nutrition transition characterized by a high consumption of fats, sweeteners and inadequate fruit intake as in the fat lovers’ cluster that was dominated by young people this study [19]. Shopping in supermarkets in Kenya is increasing and making in-roads beyond the richer consumers to lower-income groups in smaller towns with up to 56% of the customers in supermarkets reported to be from low income groups [26]. This has implications on the food choices of young people.

Education has an additional benefit in reducing NCD risk as illustrated in our study by the increase in the proportion of those in the hopeful cluster with education, however obesity increased with education. Likewise, wealth was associated with a reduction in NCD risk due to less harmful use of alcohol, salt and reduced tobacco smoking, less fat consumption but obesity also increased with wealth. Education influences health behaviors and attitudes and consequently, lifestyle through exposure to relevant health information and comprehension of the information [27]. The increase in obesity by education and wealth may be a result of increased exposure to advertisements by the food industry that has the potential to change food choices among the educated and wealthy who can afford to buy these foods.

It was also interesting to note that when three or more people shared a household, they were less likely to engage in high consumption of salt, fat, harmful consumption of alcohol and tobacco smoking. This may be largely attributed to a social audit by other household members checking on each other’s lifestyle and eating habits. For the same reason, the married are less likely to smoke or consume alcohol. Personal social networks have been reported to be associated with compliance to good health promoting behaviors [28].

Surprisingly no difference in NCD risk profile was observed between rural and urban residents contrary to studies showing that urbanicity is associated several NCD risk factors in India and Philippines [29, 30]. A recent study in rural Uganda also showed that increasing urbanicity was associated with an increase in lifestyle risk factors particularly physical inactivity, low fruit and vegetable consumption and high body mass index [31]. The common feature among these studies was the use of a multi-component scale to accurately define urbanicity even among villages considered to be rural and they found marked variation in levels of urbanicity across the villages, largely attributable to differences in economic activity, civil infrastructure, and availability of educational and healthcare services. Studies that loosely defined villages as urban or rural based on demarcation by national statistical bureaus as in this study have found no difference in NCD risk profiles among rural and urban populations, especially for hypertension [17, 18]. This suggests that even within rural populations social inequalities may exist which are often missed by the statistical bureaus because their classification of communities into rural and urban centers may not capture all the urbanicity scale components.

Regarding injuries, age, education and wealth improved compliant behaviors such as use of belts and helmets, and reduced defiant behaviors meaning as people get older or more educated or wealthier they become more responsible and tend to follow injury risk prevention measures. Education mediates comprehension of information such as written traffic rules or through an early exposure to a teaching curriculum in schools that includes traffic rules. It is worthwhile to mention that on the contrary jaywalking did not reduce with education, age, or wealth, but was instead seen to increase. A systematic review of literature on road traffic injuries in Kenya revealed that road traffic injuries have increased by four fold in three decades and up to 75% of the causalities are young adults aged 18–44 years, 80% of deaths are accounted for by pedestrians and passengers [25]. The traffic rules and enforcement seem to pay little attention to pedestrians. Most times the pedestrians break traffic rules and are not apprehended but instead treated as the victim of accidents. Public awareness about road safety especially for passengers and pedestrians is limited, thus the high risk of injuries among these groups.

The findings of this study have important implication for policy, practice and research. The identified clusters can guide where NCD policies and strategies need to focus. The resulting clusters would also be useful in the planning, implementation and evaluation of segmented approach to the prevention and control of NCDs. Similarly, future research projects could use these clusters to further explore the various characteristics associated with NCD profiles of the population of Kenya.

### Strengths and limitations

A major strength of this paper is the large sample size representative of the Kenyan population and this has provided an opportunity to investigate NCD and injury risk factors at national level. Secondly, the cluster analytical approach used in this paper identified important clusters of adult Kenyans with specific NCD and injury risk profiles for potential development of differentiated population-based interventions. However the main limitation of this cluster analytical approach is that it does not take into consideration the concurrency of risk factors, thus excludes important messages for those with multiple risk factors. Self-reported behavioural risk factors such as dietary intake and harmful use of alcohol are prone to bias, as participants may not accurately estimate quantities consumed or could purposefully conceal information for social desirability. We also excluded from the analysis individuals with incomplete records with respect to the key NCD and injury variables, which may have affected our analysis approach.

## Conclusions

In conclusion, this nationally representative survey reveals interesting patterns of NCD and injury risk clusters generated through K-medians cluster analysis which is a popular form of cluster analysis due to its simplicity of implementation, ability to partition large data sets, and ease in interpretation of its cluster solution and tolerance of outliers [32, 33]. This analysis has provided a holistic view of patterns of risk at population level for decision-makers to target populations with appropriate interventions. The main population groups to be prioritized for targeted NCD prevention interventions include; those with unhealthy diet (young fat lovers), the obese and hypertensive (older, wealthy and educated, men) and harmful users of alcohol, salt and tobacco (unmarried, older, living alone). When designing NCD preventive interventions rural populations should also be considered. Since Kenya is in the early stage of epidemiological transition, there is a window of opportunity to implement primordial NCD prevention measures to curtail the growing NCD epidemic. There is need for a multi-sectoral action to strengthen policies and implementation of programs with a focus on tackling unhealthy diet, prevention and management of hypertension and obesity. Strengthening the existing policies for tobacco and alcohol control to further reduce the current frequency of consumption and the experiences of developing these policies should inform the design of robust nutrition policies.

For injuries, there is need to design targeted messaging for road safety measures particularly for young, poor and uneducated people. Clear guidelines on safety measures for pedestrians and general public awareness on traffic guidelines for pedestrians are needed. Lastly, enhanced enforcement of traffic laws for pedestrians and passengers in public transport will be crucial in reducing road traffic injuries.

## Abbreviations

ADCA: Alcoholic Drinks Control Act
APHRC: African Population and Health Research Center
CVD: Cardiovascualr diseases
MoH: Ministry of Health, Kenya
NASSEP V: National sample surveys and evaluation programme
NCD: Non-communicable diseases
PDA: Personal digital assistant
SSA: Sub-Saharan Africa
STEPS: The WHO STEPwise approach to Surveillance of non-communicable diseases
WHO: World Health Organization
